# Trans-diagnostic determinants of psychotherapeutic treatment response: The pressing need and new opportunities for a more systematic way of selecting psychotherapeutic treatment in the age of virtual service delivery

**DOI:** 10.3389/fpubh.2023.1102434

**Published:** 2023-02-28

**Authors:** Barna Konkolÿ Thege, Talia Emmanuel, Julie Callanan, Kathleen D. Askland

**Affiliations:** ^1^Waypoint Research Institute, Waypoint Centre for Mental Health Care, Penetanguishene, ON, Canada; ^2^Department of Psychiatry, University of Toronto, Toronto, ON, Canada; ^3^Adult Psychiatric Services, Mashpee, MA, United States; ^4^Askland Medicine Professional Corporation, Midland, ON, Canada; ^5^Department of Psychiatry and Behavioural Neurosciences, McMaster University, Hamilton, ON, Canada

**Keywords:** psychotherapy, treatment selection, moderator, guideline development, tele-mental health

## Abstract

Numerous forms of psychotherapy have demonstrated effectiveness for individuals with specific mental disorders. It is, therefore, the task of the clinician to choose the most appropriate therapeutic approach for any given client to maximize effectiveness. This can prove to be a difficult task due to at least three considerations: (1) there is no treatment approach, method or model that works well on all patients, even within a particular diagnostic class; (2) several treatments are equally efficacious (i.e., more likely to be effective than no treatment at all) when considered only in terms of the patient's diagnosis; and (3) effectiveness in the real-world therapeutic setting is determined by a host of non-diagnostic factors. Typically, consideration of these latter, trans-diagnostic factors is unmethodical or altogether excluded from treatment planning – often resulting in suboptimal patient care, inappropriate clinic resource utilization, patient dissatisfaction with care, patient demoralization/hopelessness, and treatment failure. In this perspective article, we argue that a more systematic research on and clinical consideration of trans-diagnostic factors determining psychotherapeutic treatment outcome (i.e., treatment moderators) would be beneficial and – with the seismic shift toward online service delivery – is more feasible than it used to be. Such a transition toward more client-centered care – systematically considering variables such as sociodemographic characteristics, patient motivation for change, self-efficacy, illness acuity, character pathology, trauma history when making treatment choices – would result in not only decreased symptom burden and improved quality of life but also better resource utilization in mental health care and improved staff morale reducing staff burnout and turnover.

## The status quo: Psychotherapeutic treatment selection in current clinical practice

Despite the large variety of factors influencing psychotherapeutic treatment outcomes, when making treatment choices, diagnostic factors are usually prioritized when following clinical practice guidelines published by internationally recognized consortiums such as the National Institute for Health and Care Excellence (United Kingdom) (1), the Canadian Network for Mood and Anxiety Treatments (Canada) (2), and the American Psychiatric Association (United States) (3). These guidelines are disorder-specific, with most providing 1st, 2nd, and 3rd-tier treatment recommendations based on the amount of high-quality research evidence supporting the use of each individual psychotherapy modality.

Some guidelines/algorithms reference a small number of non-diagnostic factors such as severity of illness and patient preference, but to the best of our knowledge, none provide explicit direction or recommendations related to (a) how to choose from amongst treatment modalities within a single tier, (b) how to assess for and weigh non-diagnostic factors when selecting within (or across) tiers (e.g., how heavily to weigh patient preference when it opposes first-line treatment recommendations), and c) the relative importance of non-diagnostic factors in relation to treatment outcomes [cf. (4)].

Without this more detailed guidance, consideration of non-diagnostic factors is most often unmethodical, superficial, or altogether excluded from treatment plan decision-making. In the limited number of cases where these factors are indeed considered, they are typically evaluated only once a patient has been deemed “treatment-refractory” or “treatment-resistant” (5) and, therefore, at a considerable delay relative to psychotherapeutic treatment initiation. Consequences of this delay include suboptimal quality of patient care, inappropriate clinic resource utilization, patient dissatisfaction with care, patient demoralization/hopelessness, and ultimately, treatment failure.

Beyond the limitations of current, diagnosis-centered treatment guidelines, the small number of available treatment options at clinics or individual service providers has also limited the practical relevance of the question of treatment selection. Namely, in everyday clinical practice, most clinicians and (private or government-funded) mental health clinics have been able to provide a single or a very limited number of treatment approaches (6) decreasing the practical relevance of the question of how to select the best psychotherapeutic modality for their clients. That said, more careful investigation of the relative value of various treatment modalities for various patient groups may reveal that frequent practical “compromises” (e.g., offering only a single modality of treatment within a clinical setting) has the potential to be wasteful or even harmful. While common wisdom may suggest that “something is better than nothing”, this may not be the case. For example, not only do some patients become demoralized when repeatedly offered “standard” treatments but clinical staff can likewise become frustrated with patients who do not get better, contributing to potentially inappropriate discharge from care, stigmatization and safety risk.

## How did we get here? The limitations of existing treatment guidelines and their evidence base

This state of the matters presents the intriguing possibility that the very foundation of clinical practice guideline development and their use in everyday clinical practice may have a disorienting influence on treatment selection. That is, orienting practitioners to use diagnosis as the primary determinant in differential psychotherapeutic treatment consideration and selection implicitly assumes (but provides no empirical justification for) that diagnosis is the fundamental determinant of treatment response and, accordingly, should be the primary guide to psychotherapeutic treatment selection.

However, a large array of non-diagnostic factors have been proposed and/or evaluated as potential determinants (predictors, moderators and mediators) of optimal psychotherapeutic treatment selection and response [e.g., (7–10)]. Many such studies provide evidence that several non-diagnostic factors may be equally or more useful for predicting treatment response than diagnosis itself (11–22). Moreover, some of this research suggests that reliance upon diagnosis as the primary or sole basis for treatment selection may increase the probability of ineffective, inefficient, or failed treatment. Therapies that are somewhat effective under the current conditions might have significantly larger beneficial effects in terms of both specific symptom reduction and overall quality of life if matched with service users who are most responsive to the given therapeutic modality. The process of matching psychotherapeutic treatment to patient (or, for that matter, the choice of pharmacologic agents or the choice between pharmacologic and psychotherapeutic treatment approaches) involves a complex set of considerations that have been explored by various investigators over time. However, this literature is fairly siloed (23), and there appears to be little agreed-upon language that would permit this body of literature to be readily accessed and utilized by most clinicians, administrators or healthcare policy makers.

Importantly, it has also been noted that most psychotherapeutic research of the last three decades has been focused on outcomes, rather than mechanisms of action (24). This focus has the effect of reducing complex and multifactorial treatments to their labels and, in effect, entails an assumption that, for example, “cognitive behavioral therapy for panic disorder” is the same treatment across institutions, practitioners, patients, cultural contexts and time [see (20) for a more extensive discussion of this problem].

Further, the diagnosis-oriented nature of treatment guidelines is strongly influenced by the literature upon which such guideline recommendations are based: the vast majority of studies assessed when constructing clinical practice guidelines are randomized controlled trials comparing a single treatment (whether pharmacologic or psychological) to placebo or treatment-as-usual in a diagnostically homogeneous sample. Thus, there are only very few studies that could be used by guideline developers to substantiate recommendations as to the relative probability of effectiveness of one active treatment over another. Moreover, because of the difficulty in accessing (and therefore evaluating, synthesizing, and comparing) the literature on non-diagnostic factors, it is not surprising that this literature is rarely cited or systematically considered in the development of treatment guidelines and algorithms.

## A better alternative: Psychotherapeutic treatment selection systematically considering trans-diagnostic factors

Research has identified a large array of non-diagnostic factors that have been evaluated as potential determinants (predictors and moderators) of optimal psychotherapeutic treatment selection/response. When choosing among psychotherapeutic modalities, practitioners ideally should consider all or at least several of these patient-, clinician- and clinic-specific factors that can potentially impact treatment outcomes. These include – among others – sociodemographic characteristics (e.g., age, level of education, race and ethnicity), patient motivation or readiness for change, patient self-efficacy, illness acuity, specific comorbid illnesses (especially character or personality pathology), overall amount of psychopathology [cf. the p-factor (25)], trauma history, previous treatment history and outcomes, client's and clinician's preferred therapy delivery style, and clinic environment/resources.

In cases where these non-diagnosis-related factors are assessed and taken into consideration at clinic intake, patients may be more responsive to treatment (due to treatment personalization and patient engagement in treatment planning) and motivated to initiate change in emotion regulation, cognitions and behaviors. In the most ideal situation, instead of treatment assignment based on diagnoses, implicit clinician preference/bias or immediate resource availability (i.e., the typical elements influencing classic treatment selection), a set of evidence-informed predictors of treatment acceptability and response is to be used to perform personalized and holistic treatment recommendation/selection.

We anticipate that mental health treatment recipients could benefit considerably from such an evidence-based/informed systematic process for treatment selection, which would permit treatment recommendation(s) to be tailored to the individual's goals and broader characteristics predictive of treatment response. Even if no factors clearly predict a single best treatment modality (26), patients could still benefit from learning about the set of treatment modalities that are more vs. less optimal fits for their case. Moreover, a standardized (evidence-based/-informed) protocol for psychotherapeutic treatment selection could assure that the right treatment is delivered to patients who will most benefit from them (27–30), allowing for conservation of staff, clinic, and other vital mental health resources, which could also lead to improved staff morale, satisfaction with work and thus reduced staff turnover (26).

## Where do we go from here? Next steps toward more client-centered treatment selection

To remedy the suboptimal status quo, there is increased interest in applying concepts of stratified medicine in psychotherapeutic treatment selection. Stratified medicine (31) specifically seeks to refine treatment selection procedures based on identifiable moderators of differential treatment response. While the necessity of a more personalized psychotherapeutic treatment selection has likely been evident for numerous clinicians and researchers for some time (32), the large number of potential moderators compared to the relatively low number of study participants involved in efficacy and effectiveness research significantly hinders effective examination of this important issue (33).

The process of identifying and validating moderators of treatment response in mental health should ideally begin with a comprehensive and rigorous evaluation (i.e., systematic review and meta-analysis) of studies with direct comparisons of active psychological interventions in order to identify candidate factors with the best evidence as trait- or state- (34) moderators of differential treatment response (23). While there is some progress in this regard (33, 35), the conclusions of these review studies suggest that we do not yet have enough good-quality original data to inform psychotherapeutic treatment selection both because of the suboptimal investigation of moderators and the narrow range of therapeutic modalities considered in the original literature. Therefore, the allocation of dedicated resources would be essential to undertake prospective trials rigorously evaluating potential moderators that could best predict optimal treatment selection. The consideration of more psychotherapeutic approaches – including middle- and longer-term treatments as well, which have been understudied in research in the previous decades in comparison to brief, easy-to-standardize interventions – would also be necessary to make progress with the agenda of systematic treatment selection. Investigating the effectiveness of psychotherapeutic interventions on the middle and long term would also be essential to reach more reliable conclusions on which therapy should be recommended to whom (36). Comparing the effectiveness of the same therapeutic approach with matched (non-diagnostic factors also considered) vs. non-matched (only diagnosis considered) clients could help us better understand the magnitude of the difference in treatment effect (both in terms of specific psychopathological symptoms and overall quality of life) we can expect from a more systematic way of treatment selection [cf. (37)]. Finally, based on the reviewed and newly created evidence, the identified moderators should be considered when developing clinical practice guidelines and decision-aiding algorithms for systematic treatment selection in psychotherapeutic practice.

We believe that the seismic shift toward virtual psychotherapeutic service delivery due to the COVID pandemic – despite the numerous challenges – offers a huge opportunity to move toward more systematic treatment selection; both in terms of generating research evidence and allowing a more client-centered clinical practice. With virtual service delivery, the limitations of a given clinician or particular clinic now pose significantly smaller barriers than in the past as more distant service providers with a better match to client characteristics have recently become realistic alternatives. While it may be true that certain client populations [most likely those with more severe pathology cf. (38)] are less suitable for online service delivery, we believe that the vast majority of psychotherapy recipients with mild to moderate level of functional difficulties can benefit similarly from virtual/online vs. face-to-face psychotherapy (39, 40).

We argue that the shift toward virtual service delivery could also bring new opportunities *via* (1) online services that offer help to treatment seeking individuals in finding mental health service providers^1^ and (2) platforms offering online outcome monitoring services to a large number of diverse mental health clinicians^2^ (this could also work in conjunction with traditional, face-to-face therapy delivery). These organizations – which already collect a large amount of client data, including both real-life outcome data and potentially relevant moderator variables – in collaboration with researchers, could easily collect and analyze a vast amount of data on client characteristics and treatment outcomes. These data, in turn, could facilitate the development of algorithms to support more optimal treatment selection, improving the chance of success for each client (and their treatment provider).

Further, mid-sized or large mental health care organizations could now expand the range of therapeutic approaches available within their systems in a financially feasible way and match clients to the most promising treatment approach regardless of the physical distance between client and therapist. While investing in the training of staff in therapeutic modalities ideal to less (but still a significant number of) clients was not feasible in the past, the current landscape of online service delivery allows organizations or clinician networks to assess and diversify the therapeutic modalities available within their systems and use them in an economic way for the benefits of all (not just the assumed or actual majority of) clients, therapists, and the mental health care system as a whole.

## Conclusion

Psychotherapeutic treatment selection is a largely neglected topic within the mental health care literature. Given that diagnosis alone is insufficiently predictive of psychotherapeutic treatment outcome, it is clear that non-diagnostic factors contribute to differential effectiveness and efficiency. Despite this fact, clinical practice guidelines are organized entirely around diagnosis and rarely reference non-diagnostic factors in recommending or prioritizing treatment options. We propose that this diagnostically-oriented framework for psychotherapeutic treatment selection omits critical patient-, therapist- and clinic/contextual factors that could help increase the overall effectiveness of psychotherapy, which – some argue – are much more limited (36, 41) or actually, more harmful [cf. (42, 43)] in the real-life setting than many strictly controlled trials indicate. Moreover, failure to account for non-diagnostic factors likely contributes to treatment misapplication, clinical waste and, perhaps, avoidable harm to patients and staff morale. We propose that a systematic, research-based consideration of non-diagnostic factors in psychotherapeutic treatment selection is desirable and possible. The COVID pandemic has facilitated the use of and comfort with online service delivery both in treatment recipients and providers. Thus, while geographic proximity had long been a limiting factor in patient access to best-matching psychotherapeutic care, it should no longer serve as a justification for a “one-size-fits all” approach to psychotherapeutic treatment availability and selection.

## Data availability statement

No datasets were generated for this study.

## Author contributions

KA developed the concept of the paper. KA and BKT wrote the first draft of the manuscript. All authors contributed to reviewing and interpreting the extant literature, final version as well as read, and approved the submitted version.

## Footnotes

^1^For example: https://www.betterhelp.com or https://www.psychologytoday.com/ca/therapists. Please note that these are simply examples to allow readers to consider the authors' argumentation more in-depth. Mentioning these services means no endorsement of these companies by the authors for any reason.

^2^For example: https://www.myoutcomes.com or https://www.greenspacehealth.com/en-ca/. Please note that these are simply examples to allow readers to consider the authors' argumentation more in-depth. Mentioning these services means no endorsement of these companies by the authors for any reason.
